# 

**Published:** 1877-01

**Authors:** 


CONTENTS.

| | Page | | Page |
|---|---|---|---|
| Consumption | 1 | To Bring Up Children | 17 |
| Drinking at Meals | 3 | Never | 18 |
| Politics and Physic | 5 | Healthy Employments | 19 |
| Still They Come | 8 | Throat-Ail Causes | 21 |
| Cold Baths | 11 | To be Safe | 21 |
| Slandering Doctors | 13 | Miscellaneous | 22 |

